# Diabetes-related quality of life in six European countries measured with the DOQ-30

**DOI:** 10.1080/13814788.2021.1954615

**Published:** 2021-08-02

**Authors:** Liina Pilv, Etienne I. J. J. Vermeire, Anneli Rätsep, Alain Moreau, Davorina Petek, Hakan Yaman, Marje Oona, Ruth Kalda

**Affiliations:** a Institute of Family Medicine and Public Health, University of Tartu, Tartu, Estonia; b Faculty of Medicine and Health Sciences, University of Antwerp, Antwerp, Belgium; c Department of Family Medicine, University Claude Bernard Lyon, Lyon, France; d Department of Family Medicine, Faculty of Medicine, University of Ljubljana, Ljubljana, Slovenia; e Akdeniz University, Antalya, Turkey

**Keywords:** Type 2 diabetes (T2DM), patients with diabetes (PWD), diabetes-related quality of life (DR-QoL), the Diabetes Obstacles Questionnaire-30 (DOQ-30)

## Abstract

**Background:**

The quantification of diabetes-related quality of life (DR-QoL) is an essential step in making Type 2 Diabetes (T2DM) self-management arrangements. The European General Practitioners Research Network (EGPRN) initiated the EUROBSTACLE study to develop a broadly conceptualised DR-QoL instrument for diverse cultural and ethnic groups; high and low-income countries. In 2016 the Diabetes Obstacles Questionnaire-30 (DOQ-30) was introduced.

**Objectives:**

The research aimed to study obstacles a patient with diabetes (PWD) may face in everyday life. First, we assessed how descriptive and clinical characteristics and the residential country were associated with the obstacles. Secondly, we calculated the proportion of respondents who expressed obstacles.

**Methods:**

Data were collected in 2009 in a cross-sectional survey in Belgium, France, Estonia, Serbia, Slovenia, and Turkey. Multiple linear regressions were computed to detect associations between descriptive and clinical characteristics, residential country, and obstacles. Percentages of respondents who perceived obstacles were calculated.

**Results:**

We found that although descriptive and clinical characteristics varied to quite a great extent, they were weakly associated with the perception of obstacles. The residential country was most often associated with the existence of some obstacle. The highest percent (48%) of all respondents perceived ‘Uncertainty about Insulin Use’ as an obstacle.

**Conclusion:**

Descriptive and clinical characteristics were weakly associated with perceived obstacles. However, the residential country plays an essential role in the decline of the QoL of PWDs. Education of both PWDs and healthcare professionals (HCPs) plays an essential role in countering the fear of insulin.

KEY MESSAGES

- PWDs perceived obstacles in every country: in high and low-income, western and eastern European countries, and Turkey.
- Astonishing, the most substantial obstacle in five of six countries was ‘Uncertainty about Insulin-Use’.
- The DOQ-30 is a valuable instrument to find out obstacles in everyday life with diabetes.

## Introduction

Type 2 Diabetes (T2DM) is a chronic, progressive disease that requires adherence to treatment recommendations, self-management, and lifestyle alterations. It can cause stress in everyday life for T2DM, culminating in a decrease in diabetes-related quality of life (DR-QoL) and a rise in haemoglobin A1c (HbA1c) [1–4]. In 2000, the EGPRN initiated a project to develop a usable DR-QoL instrument to assess obstacles in the everyday lives of PWD, which was internationally diverse to racial, cultural, and ethnic groups and which included low-income countries. In the first stage, the qualitative EUROBSTACLE study was carried out, resulting in the establishment of the DOQ comprised of 78 items [5]. A cross-sectional study with the DOQ was conducted from May to November 2009 in six countries: Belgium, France, Estonia, Serbia, Slovenia, and Turkey. The DOQ was validated in England, Belgium, and Estonia [6–8]. In the second stage of the project, the DOQ underwent exploratory factor analysis (EFA). The DOQ-30, a short version comprised of 30 items, was then created. The DOQ-30 showed good to excellent correlation with the DOQ and demonstrated good internal, external, and construct validity for the study's whole sample [9].

Studies have shown that a PWD’s country of residence significantly impacts DR-QoL [10,11].

This article aims to study how PWD perceived obstacles by using the DOQ-30 in different countries. Our cross-sectional survey includes some rich western European countries, such as France and Belgium; less wealthy post-Soviet eastern European countries, such as Estonia, Serbia, Slovenia, and Turkey, situated between Europe and Asia. At first, we compare the differences between descriptive characteristics and health outcomes and investigate whether the obstacles were related to it and the residential country. The second aim is to find out how many respondents in each country perceived obstacles in everyday life.

## Methods

### The dataset

Data for the study were collected from May to November 2009 in a cross-sectional survey in six European countries: Belgium, France, Estonia, Serbia, Slovenia, and Turkey. GPs enrolled at least three consecutive outpatient PWDs in the sample. We used data from 853 respondents for the statistical analyses. The dataset consists of PWDs’ responses to the DOQ-30 and descriptive and clinical characteristics from the participants’ medical records sent by their GPs [6–9].

### The instrument of study

In the study, we used the DOQ-30, which was developed by exploratory factor analyses (EFA) from the DOQ [9]. The DOQ-30 is a measure of DR-QoL in nine obstacle scales, and it comprises 30 items. Each scale pertains to one theme of DR-QoL. The questions were scored on a five-point Likert scale: eight negatively-worded items ranging from 0 (Strongly Agree, continuously present) to 4 (Strongly Disagree, not present), and one positively-worded item ranging from 0 (Strongly Disagree) to 4 (Strongly Agree).

### Scoring of the DOQ-30 instrument

The five-point Likert scale fluctuated between 0 and 4. We standardised this to a score from 0 (best thinkable well-being, having no obstacles) to 1 (worst thinkable well-being).

A cut-off point with a value of 0.5 meant ‘neutral’, which is rated as ‘no obstacle’. A result above 0.5 indicated that the respondent perceived an obstacle in the corresponding theme of the DOQ-30. Almost the same methodology was used in the MIND study [12].

### Statistical analysis

####  

##### Descriptive and clinical statistics of the study sample

Frequency and percentages were calculated for descriptive categorical characteristics related to gender, type of diabetic treatment, and smoking status. We studied mean and standard deviation (SD) for all quantitative variables, such as age, T2DM duration, weight, height, BMI, and disease-related variables, such as haemoglobin A1c (HbA1c), total cholesterol (Chol), systolic blood pressure (Syst-BP), diastolic blood pressure (Diast-BP).

###### Association of obstacles with residential country, descriptive and clinical characteristics

We calculated multiple linear regressions (MLR) with *p*-value to analyse associations between the residential country, descriptive characteristics, and clinical outcomes of the PWD on the one hand and the obstacles listed in the DOQ-30 on the other. The strength of the association was characterised by regression coefficient *β*.

###### Proportions of respondents who perceived obstacles

We calculated the percentage of respondents in each country who answered that they perceived obstacles at a value above ‘neutral’. To illustrate how many respondents in the participating country reported obstacles, we drew bar charts. To analyse all the nine themes of the DOQ-30 we drew point plot graphs with 95% CI indicating the percentage of respondents who perceived the theme as an obstacle. We used the most conservative Spearman-Clopper method to test two-sided confidence intervals for the single dimension [13].

The statistical analysis was carried out using R-statistics, version 4.0.0, and IBM SPSS Statistics, version 24.

## Results

### Descriptive and clinical statistics of the study sample

First, we present descriptive data on the enrolled PWD. Some of these data have also been published previously [9].

Descriptive characteristics in the study group of 853 participants showed considerable variation between countries. The participants’ age ranged from 27 to 89 years; the oldest PWDs were in Estonia and the youngest in Turkey. The highest number of pill-takers and the lowest number of insulin-users lived in Turkey. HbA1c is a crucial feature to follow up and examine for associations with DR-QoL. The mean level of HbA1c of all study participants was 7.4 (*SD* 1.4). The lowest mean level of HbA1c was in Belgium and Estonia (7.1), and the highest was in Turkey (8.2). All descriptive characteristics of the whole sample are presented in Table 1.

**Table 1.** Descriptive and clinical statistics of study sample for participating countries.

	Country						
	Estonia	France	Serbia	Slovenia	Turkey	Belgium	Total
Age in years, mean (*SD*)	66.7 (9.8)	65.0 (9.5)	64.2 (10.3)	63.0 (10.1)	59.3 (11.3)	65.6 (10.4)	64.1 (10.5)
T2DM duration in years, mean (*SD*)	8.6 (5.0)	10.2 (8.1)	11.0 (7.3)	9.7 (6.6)	3.7 (3.0)	1.6 (1.3)	7.3 (6.7)
Gender male, *n* (%)	61 (44.5)	105 (58.3)	50 (45.0)	74 (57.4)	57 (41.0)	76 (48.4)	423 (49.6)
Tablets treatment, *n* (%)	124 (90.5)	162 (89.5)	94 (84.7)	90 (75.6)	130 (93.5)	137 (87.8)	737 (87.4)
Insulin treatment, *n* (%)	38 (29.0)	45 (24.9)	43 (38.7)	28 (23.5)	24 (17.3)	47 (30.3)	225 (26.9)
Smoking, *n* (%)	23 (17)	29 (16)	22 (20)	10 (8)	29 (21)	21 (13)	134 (16)
BMI in kg/m^2^, mean *(SD)*	32.5 (6.0)	31.2 (6.2)	27.3 (3.5)	30.9 (5)	30.4 (6.2)	29.6 (5.5)	30.4 (5.7)
HbA1c, mean (*SD*)	7.1 (1.2)	7.3 (1.2)*	7.4 (1.4)	7.5 (1.4)	8.2 (1.8)	7.1 (1.1)	7.4 (1.4)
CHOL (mmol/L), mean (*SD*)	5.2 (1.1)	4.6 (1.2)	5.6 (1.0)	4.7 (1.1)	5.3 (1.2)	4.7 (0.9)	5.0 (1.2)
Syst-BP (mmHg), mean (*SD*)	138.8 (11.5)	133.4 (11.3)	135.3 (14.2)	135.7 (14.7)	137.4 (15)	134.3 (14.6)	135.7 (13.6)
Diast-BP (mmHg), mean (*SD*)	82.7 (8.5)	77.9 (7.8)	81.8 (7.6)	78.8 (9.1)	82.4 (8.4)	77.7 (9.5)	80 (8.8)
Total, *n* (%)	137 (100.0)	180 (100.0)	111 (100.0)	129 (100.0)	139 (100.0)	157 (100.0)	853 (100.0)

### Associations of obstacles with residential country, descriptive and clinical characteristics

The ratings of characteristics connected with any theme in the DOQ-30 are presented in Table 2. We found that the country of residence was significantly associated with the perception of some obstacles. Regression coefficients *β* extended from 0.085 to 0.369. PWD who lived in Turkey perceived five obstacles out of nine more intensively than the mean of the total study sample. On the contrary, the level of HbA1 was associated with all themes, though this association was weak (*β* 0.016–0.031). Non-usage of insulin treatment negatively affects (*β* − 0.177) confidence in insulin. Some other variables had a statistically low impact and showed a regression coefficient *β* from 0.002 to 0.177.

**Table 2.** Associations of obstacles with residential country, descriptive and clinical characteristics.

	Regression coefficient *β*/*p*-value *p < 0.05; **p < 0.005; ***p < 0.001								
	OBS-1	OBS-2	OBS-3	OBS-4	OBS-5	OBS-6	OBS-7	OBS-8	OBS-9
Country									
Estonia		0.095**	0.114**		0.134***	0.093*			
France						0.107**		−0.085*	
Serbia					0.169***		−0.143***		
Slovenia						0.120**		−0.113**	
Turkey	0.187***	0.129***	0.154***		0.266***	0.180***			
Belgium									0.369***
Age		−0.002*	0.002*				−0.002*		
T2DM duration			−0.004*			−0.005**			
Gender = M							−0.048**		
Tablets = YES		−0.052*							
Insulin = YES									−0.177***
Smoking = YES					0.064*				
BMI					0.007***				
HbA1c	0.024***	0.018**	0.031***	0.027***	0.019**	0.029***	0.024***	0.026**	0.016*
Chol			0.019*						
Syst-BP									
Diast-BP									
Total, *n*	679	668	658	674	674	551	678	664	651
*R* squared	0.16	0.121	0.145	0.098	0.208	0.133	0.1	0.061	0.288

OBS-1: obstacles in relationships with healthcare professionals; OBS-2: feeling alone and deficiency of social support; OBS-3: shortage of knowledge about diabetes; OBS-4: obstacles associated with changes in diet and lifestyle; OBS-5: obstacles associated with exercising; OBS-6: obstacles associated with self-monitoring; OBS-7: uncertainty about consultation; OBS-8: uncertainty about diabetes medication; OBS-9: uncertainty about insulin-use.

Blank cells indicate that there were no significant associations between characteristics and obstacle scales.

### Proportions of respondents who perceived obstacles

**Figure 1.**
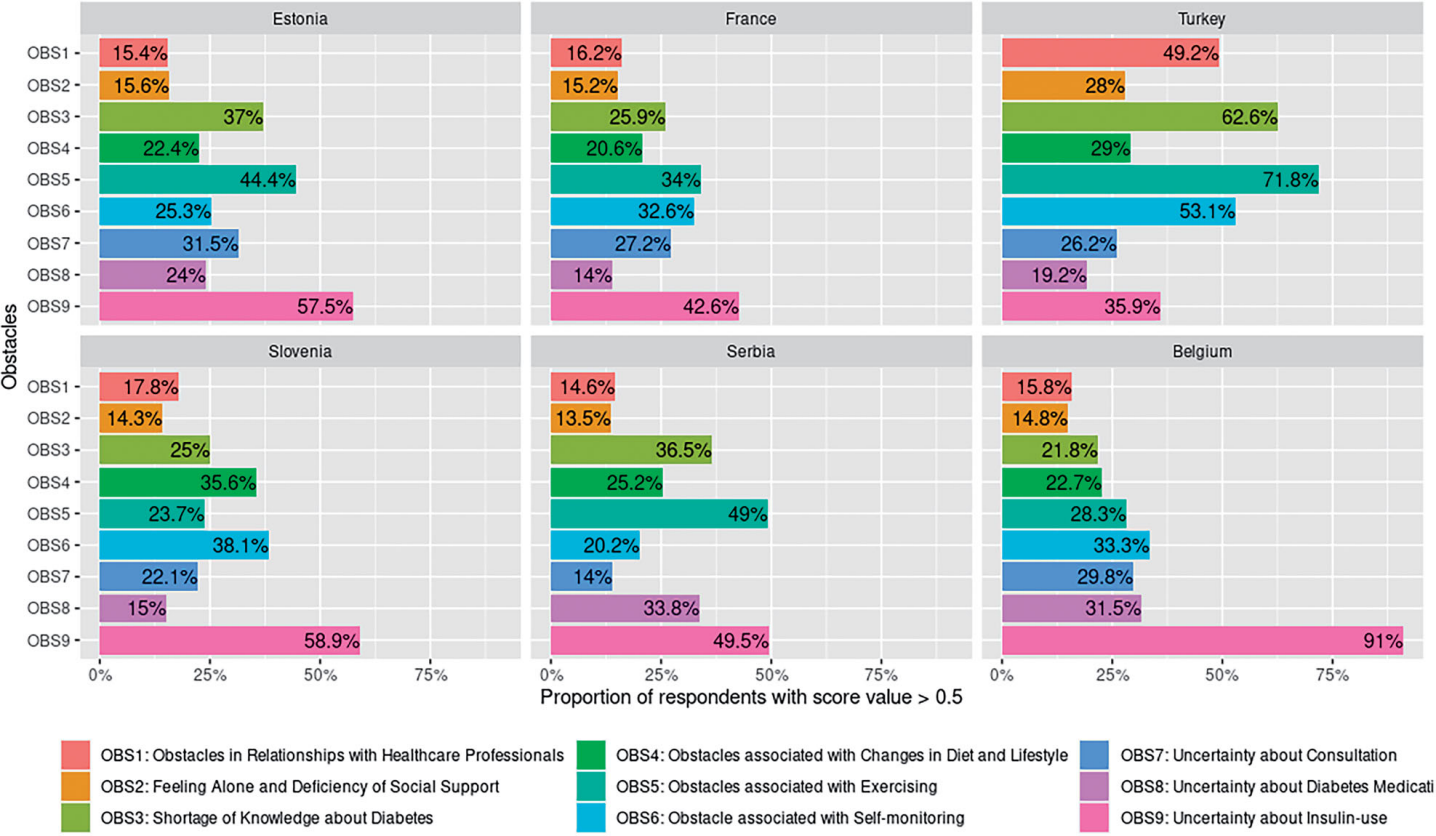
Proportions of patients with diabetes who perceived obstacles.

These are shown in Figure 1. The analysis revealed that respondents from Turkey perceived more obstacles than others did.

More than 50% of respondents recognised three themes (knowledge, exercise, and self-monitoring) as obstacles. More than half of Belgian and Estonian respondents expressed uncertainty about insulin use. To illustrate how many respondents reported increased pressure from the obstacle and compare this to the mean of dissatisfied participants of the whole study sample, we drew point plot graphs presented in Charts 1–9 (available as Supplemental Material online).

## Discussion

### Main findings

Successful diabetes care requires supporting patients’ efforts to change behaviour about the obstacles that suppress the DR-QoL [14]. We investigated obstacles with the DOQ-30 in Belgium, France, Estonia, Serbia, Slovenia, and Turkey. According to their descriptive and clinical features, patients with diabetes in all these countries were somewhat incompatible, which minimally influenced their perception of obstacles. PWDs in all countries reported obstacles, despite quite notable differences between the countries. The percentage of PWDs dissatisfied with some aspect of DR-QoL was highest in Turkey. Uncertainty about insulin use and obstacles associated with exercising were the greatest impediments in everyday life with diabetes.

### The limitations and strengths of the present study

We acknowledge the limitations of this study, namely that it was conducted in 2009. The results do not reflect a contemporary DR-QoL but that of 2009. A second limitation is that the study sample of respondents in the countries was relatively small, ranging from 111 to 180. Third, there were some essential social, occupational, educational, cultural, and religious characteristics that we did not assess. Nevertheless, perceived obstacles in living with diabetes remain a perennial issue. We have claimed that the DOQ-30, developed by our working group, is a broadly conceptualised instrument for detecting obstacles in diverse racial, cultural, and ethnic groups and high as well as low-income countries. Although the data was collected in earlier years, detecting and measuring DR-QoL on a widespread basis remains a worthwhile undertaking. This instrument was first successfully used in Africa [15].

### The interpretation of the study results in relation to literature

Our study found considerable differences in descriptive variables, such as age, duration of T2DM, type of diabetic treatment, and disease-related variables, such as HbA1c and total cholesterol (Table 1).

Gender, age, and duration of the disease correlated unconvincingly with obstacles (Table 2). Furthermore, in the literature, authors have expressed contradictory views on these topics. As in some other studies [12,16,17], females in ours perceived slightly more obstacles than males. However, other researchers found no significant gender differences [18]. In total, 48% of all participants who were on non-insulin treatment and 91% of Belgians (Figure 1, Chart 9), for whom T2DM had lasted ∼1.6 years (Table 1), expressed anxiety about insulin use. In other studies, PWDs expressed uncertainty about diabetic medication: older patients about sulphonylurea [19] and younger patients about insulin [20]. The diabetic medication interferes negatively with these patients` ideas about how they want to live their everyday lives [8,21,22]. In contrast, a significant number of PWDs not receiving insulin in Japan were prepared to start injections if they were prescribed [23]. We may be seeing a theme of knowledge about the disease here.

Higher HbA1c reduced contentment in every topic area: perceived obstacles in relationships with healthcare professionals (HCPs), shortage of diabetes-related knowledge, self-testing problems, and difficulties with lifestyle changes (Table 2). In general agreement with the literature cited, inadequate metabolic control of blood sugar level was independently associated with poorer well-being of PWDs [17,24,25]. Similar to ours, some studies claim that HbA1c has a weak relationship with QoL [26].

As we assumed, the number of experienced obstacles differed among participating countries.

We found support in the literature for this outcome [27,28]: PWDs in Canada and Denmark assessed their psychosocial QoL to be good or very good [29,30], but 41% of respondents in France affirmed some negative impact of their disease on physical, emotional and leisure activities [31]. In our study, up to 42.6% of French and up to 58.9% of East-European (Estonia, Serbia, and Slovenia) respondents expressed any of the nine obstacles (Figure 1, Chart 1–9). Turkey’s respondents revealed above-average dissatisfaction with five out of nine issues (Figure 1, Chart 1–9). Problems related to obstacles in Turkey were consistent with previous studies [32] and may partly be due to cultural differences [33].

Obstacles concerning dietary changes and strains on social relationships were the most negatively rated QoL aspects in Denmark, the Netherlands, the UK, Singapore, and Australia [17,25]. In our study, only 35.6% of Slovenian and 29% of Turkish respondents perceived the same obstacles to a greater extent than the study mean value (Figure 1, Chart 4). In the WHO report, the prevalence of physical inactivity is highest in low-income countries, almost double that in high-income countries [10]. In our study, 44% of Estonians, 49% of Serbians, and 71.8% of Turkish PWD experienced obstacles concerning participating in physical activities (Figure 1, Chart 5). Patient-centred care presumes excellent communication with HCPs, comfortable consultation, and PWD's knowledge and understanding of T2DM. Krass et al. [2] claimed that patients who reported high decision-making involvement with HCPs were more likely to have better adherence to diabetes management. In our study, 49.2% of Turkish PWDs and up to 24% of all other countries reported hindrances in communications with HCPs (Figure 1, Chart 1). The first study with the DOQ-30 questionnaire was carried out in northwest Ethiopia, concluding that there were obstacles related to PWDs’ relationship with HCPs, lack of support from their friends, lack of knowledge about T2DM, and lack of motivation to exercise [15]. This pattern of barriers was very similar to Turkey. Peyrot et al. [20] and Nicolucci et al. [34] hypothesised that adherence to diabetic medication might vary across countries due to cultural and medical or healthcare systems differences. Vermeire et al. [5] were reticent concerning the opposite standpoint, claiming that patients' beliefs, attitudes, knowledge about diabetes, and their relationships with healthcare professionals were significant. In our study, older respondents reported slightly more significant insufficiency in learning about diabetes (Table 2). A shortage of knowledge and uncertainty about the benefits of diabetic medication could culminate in low adherence to treatment while also pointing to the value of a good relationship between PWDs and HCPs.

## Conclusion

Patients' obstacles to treatment continue to require careful assessment, and research on DR-QoL continues to be relevant. We studied nine different themes of diabetes-related quality of life in six European countries and found that descriptive and clinical characteristics, including HbA1c, are weakly associated with perceived obstacles. The strongest influencing factor was residential country. Turkey's respondents expressed frustration to five out of nine obstacles. The most decisive obstacle claimed is fear of insulin use. In today's clinical practice, an approach centring on and involving patients is essential to achieve the best treatment outcome and better patient self-management.

## Supplementary Material

The Diabetes Obstacles Questionnaire -30 (DOQ-30)

OBSTACLES Charts 1-9
