# Supplementary material for: Diabetes-related quality of life in six European countries measured with the DOQ-30
Source: Eur J Gen Pract. 2021 Aug 2;27(1):191–7. doi: 10.1080/13814788.2021.1954615 (PMC8330755; doi:10.1080/13814788.2021.1954615)
Supplement: The Diabetes Obstacles Questionnaire -30 (DOQ-30) [file IGEN_A_1954615_SM1177.pdf]

The Diabetes Obstacles Questionnaire -30 (the DOQ-30)

The DOQ-30 — a DR-QoL questionnaire

Please place one tick against each comment to indicate how much you agree or disagree with the statement with your **DISTRESS level related to diabetes.**

L. PILV ET AL. liina.pilv@ut.ee

OBS-1	Obstacles in Relationships with Healthcare Professionals	Stongly Agree	Agree	Neutral	Disagree	Strongly Disagree
1	I am not assisted in setting realistic targets for changing my lifestyle.					
2	Treatment alternatives are not explained to me.					
3	I have not been told what to expect from my treatment.					
4	The good and bad aspects of each choice have not been discussed with me.					

OBS-2	Feeling Alone and Deficiency of Social Support	Stongly Agree	Agree	Neutral	Disagree	Strongly Disagree
5	I feel I get little support from my family.					
6	I feel I get little support from my friends.					
7	I feel very alone with my diabetes.					
8	I would manage my diabetes much better if I had encouragement socially.					

OBS-3	Shortage of Knowledge about Diabetes	Stongly Agree	Agree	Neutral	Disagree	Strongly Disagree
9	I have difficulty understanding the information from the literature.					
10	I do not know as much as I need to know to manage my diabetes.					
11	I do not know as much as I need to know about the consequences of having diabetes.					
12	I do not know enough about the treatment of diabetes.					

OBS-4	Obstacles associated with Changes in Diet and Lifestyle	Stongly Agree	Agree	Neutral	Disagree	Strongly Disagree
13	My diabetes has placed a strain on my personal relationships.					
14	Changes in my diet have put a strain on my family.					
15	I feel resentful that I am obliged to change my eating habits.					
16	My diabetic diet spoils my social life.					

OBS-1	Obstacles in Relationships with Healthcare Professionals	Stongly Agree	Agree	Neutral	Disagree	Strongly Disagree
**OBS-5**	**Obstacles associated with Exercising**	Stongly Agree	Agree	Neutral	Disagree	Strongly Disagree
17	I have not found an exercise I enjoy.					
18	I lack the motivation to exercise.					
19	I am unable to fit exercise into my lifestyle.					
20	I am unable to afford the cost of exercising regularly.					
**OBS-6**	**Obstacle associated with Self-monitoring**	Stongly Agree	Agree	Neutral	Disagree	Strongly Disagree
21	Self-monitoring makes me feel frustrated.					
22	I find it too uncomfortable to self-monitor.					
23	I find it especially hard to test when I am busy.					
24	Self-monitoring makes me fearful of a high reading.					
**OBS-7**	**Uncertainty about Consultation**	Stongly Agree	Agree	Neutral	Disagree	Strongly Disagree
25	I feel a sense of helpless when consulting with nurses.					
26	The way that I was told that I had diabetes made me feel afraid.					
**OBS-8**	**Uncertainty about Diabetes Medication**	Stongly Agree	Agree	Neutral	Disagree	Strongly Disagree
27	I do not feel I am being prescribed a medication that is right for me.					
28	I do not feel I am being prescribed a medication dose that is right for me.					
**OBS-9**	**Uncertainty about Insulin-use**	Stongly Agree	Agree	Neutral	Disagree	Strongly Disagree
29	Using insulin makes life too complicated.					
30	Using insulin means my diabetes is getting worse.					
