# Supplementary material for: Diabetes-related quality of life in six European countries measured with the DOQ-30
Source: Eur J Gen Pract. 2021 Aug 2;27(1):191–7. doi: 10.1080/13814788.2021.1954615 (PMC8330755; doi:10.1080/13814788.2021.1954615)
Supplement: OBSTACLES Charts 1-9 [file IGEN_A_1954615_SM1176.pdf]

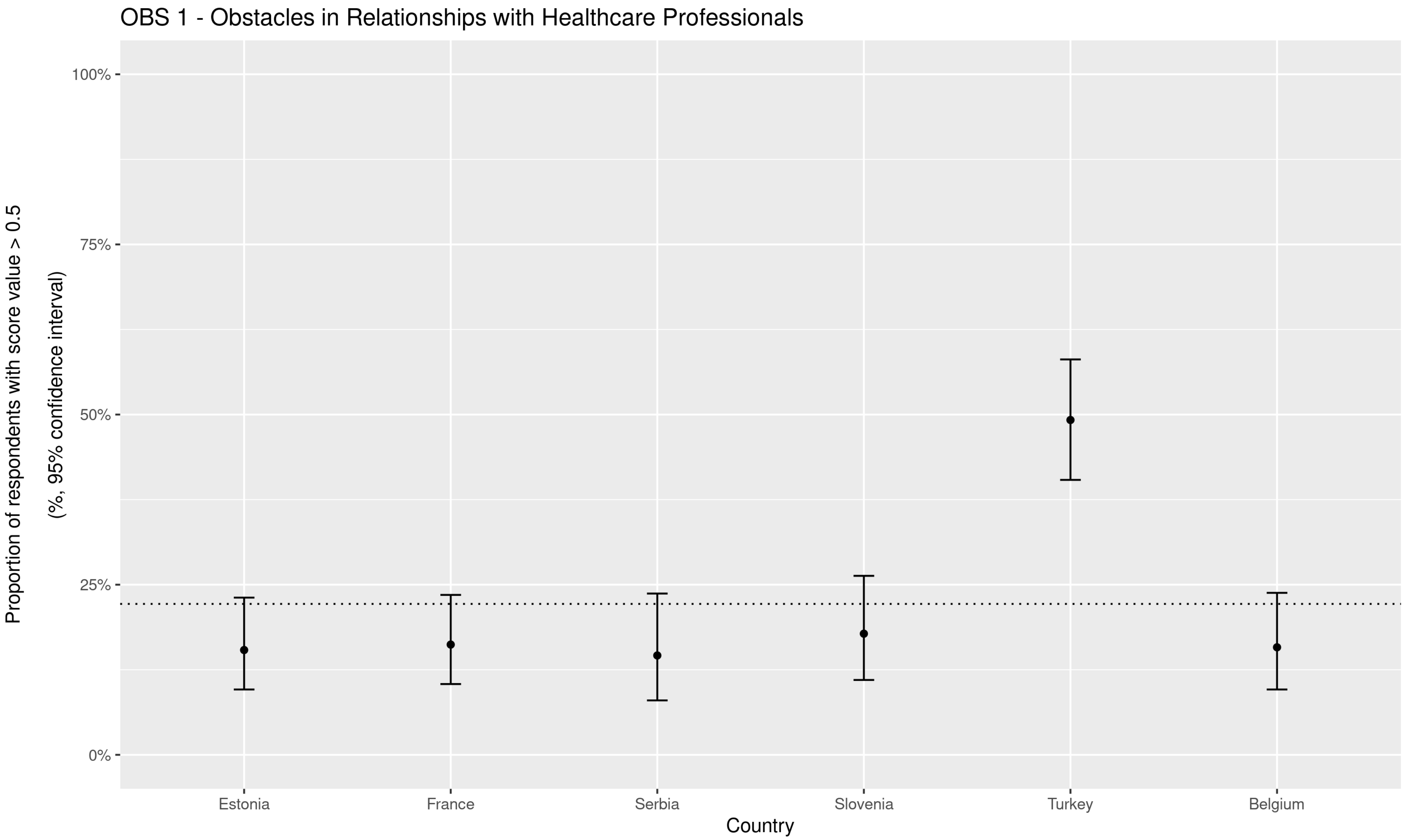
OBS 1 - Obstacles in Relationships with Healthcare Professionals
Proportion of respondents with score value > 0.5
(%, 95% confidence interval)
100%
75%
50%
25%
0%
Estonia
France
Serbia
Slovenia
Turkey
Belgium
Country

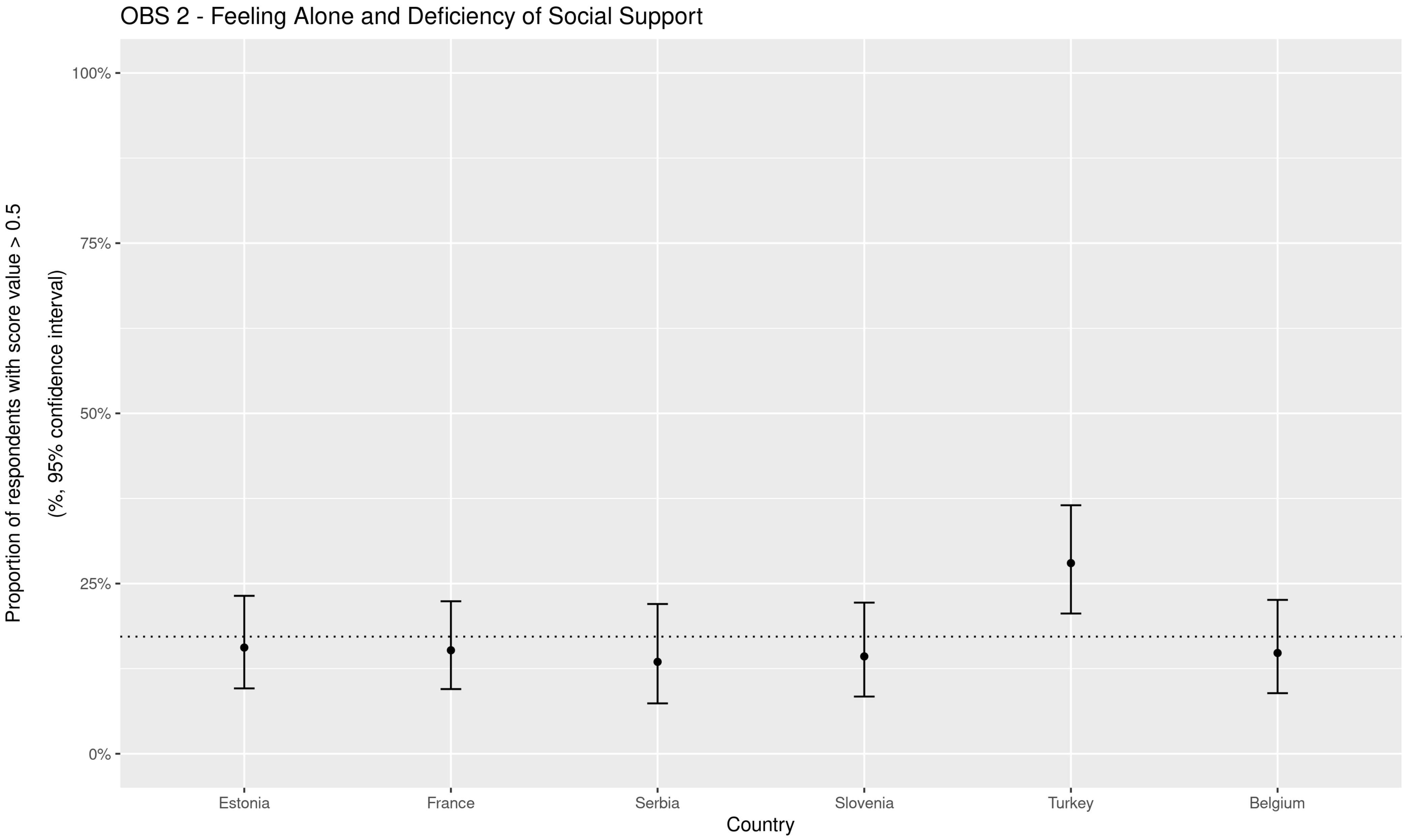

OBS 2 - Feeling Alone and Deficiency of Social Support
Proportion of respondents with score value > 0.5
(%, 95% confidence interval)
100%
75%
50%
25%
0%
Estonia
France
Serbia
Slovenia
Turkey
Belgium
Country

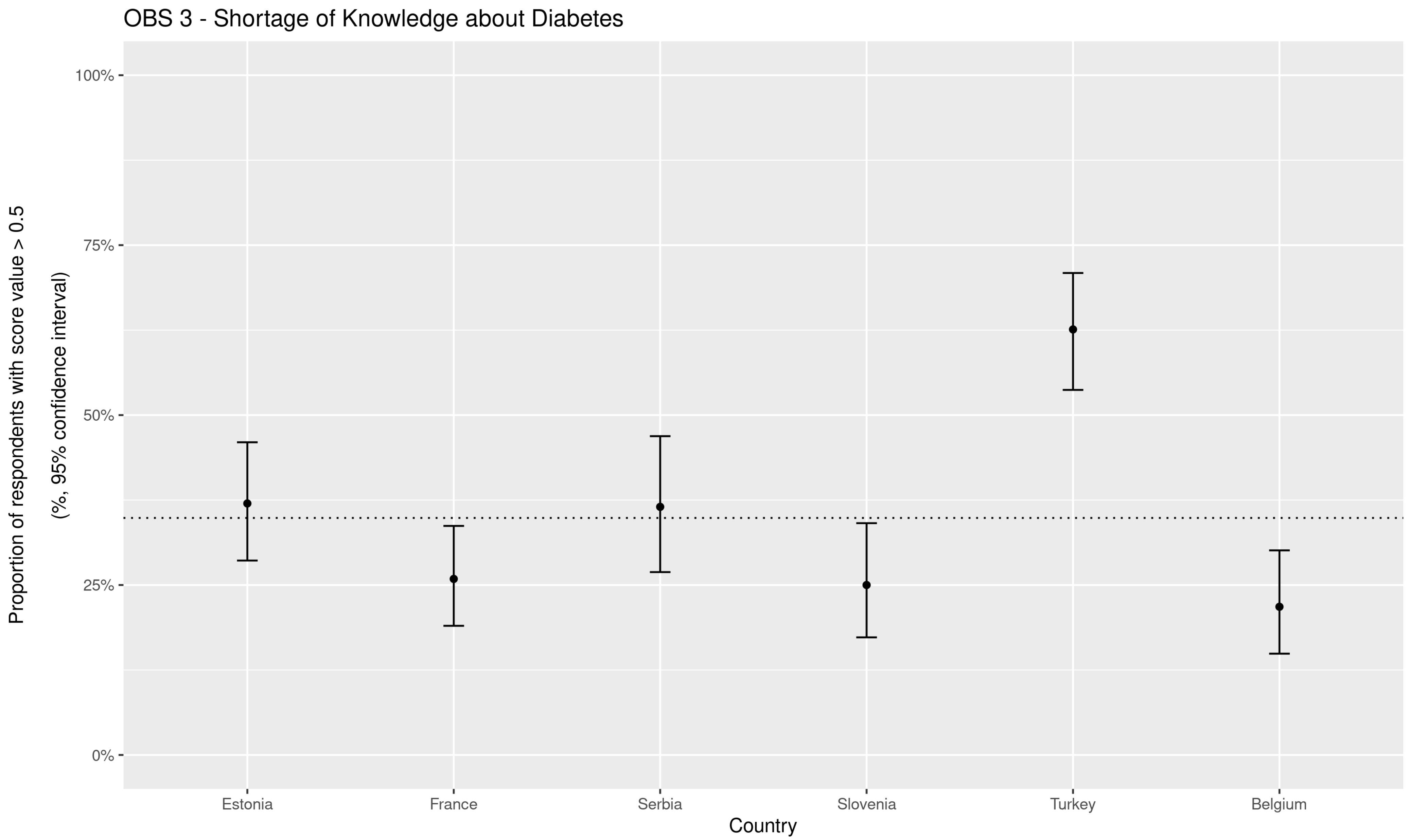

OBS 3 - Shortage of Knowledge about Diabetes
Proportion of respondents with score value > 0.5
(%, 95% confidence interval)
100%
75%
50%
25%
0%
Estonia
France
Serbia
Slovenia
Turkey
Belgium
Country

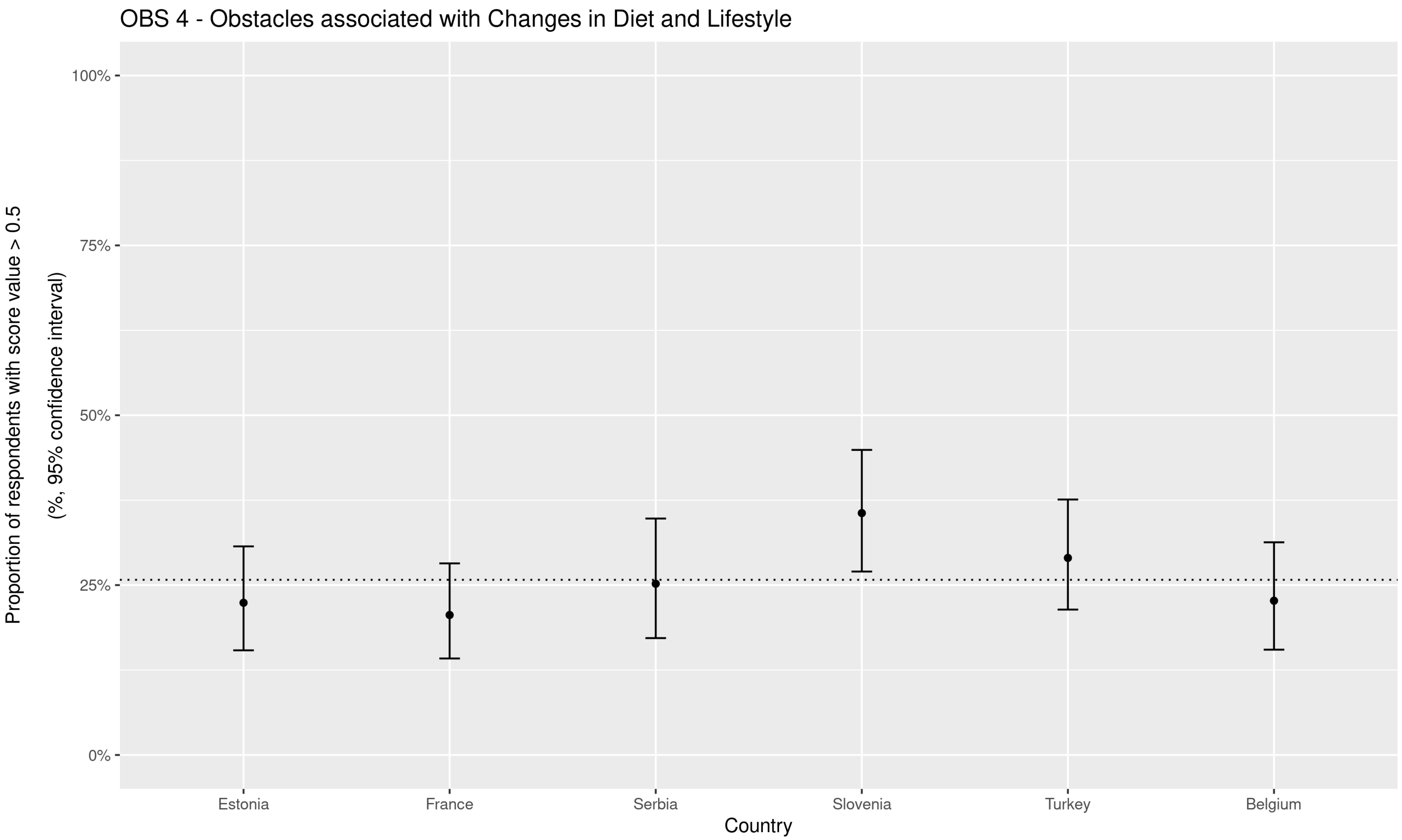

OBS 4 - Obstacles associated with Changes in Diet and Lifestyle
Proportion of respondents with score value > 0.5
(%, 95% confidence interval)
100%
75%
50%
25%
0%
Estonia
France
Serbia
Slovenia
Turkey
Belgium
Country

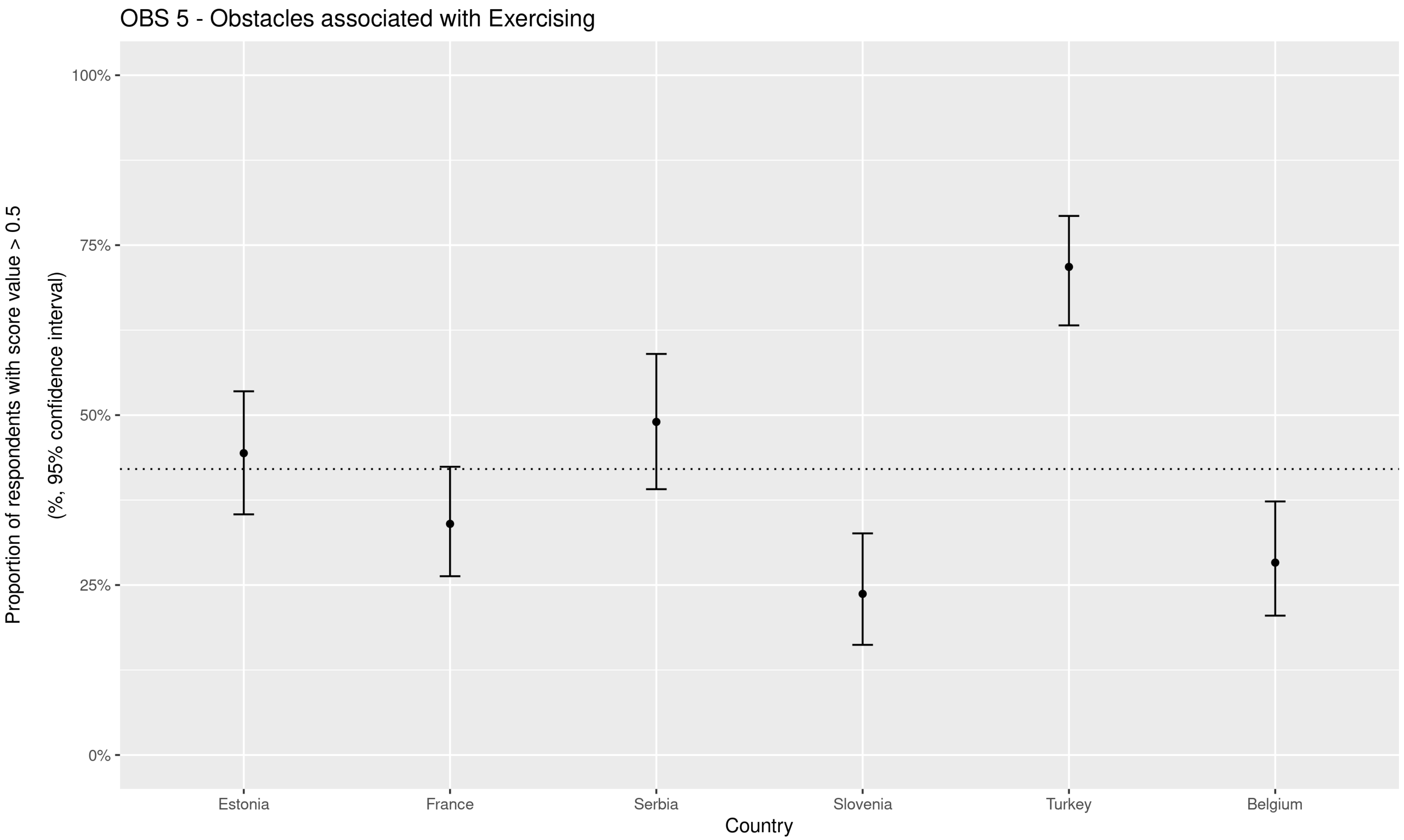

OBS 5 - Obstacles associated with Exercising
Proportion of respondents with score value > 0.5
(%, 95% confidence interval)
100%
75%
50%
25%
0%
Estonia
France
Serbia
Slovenia
Turkey
Belgium
Country

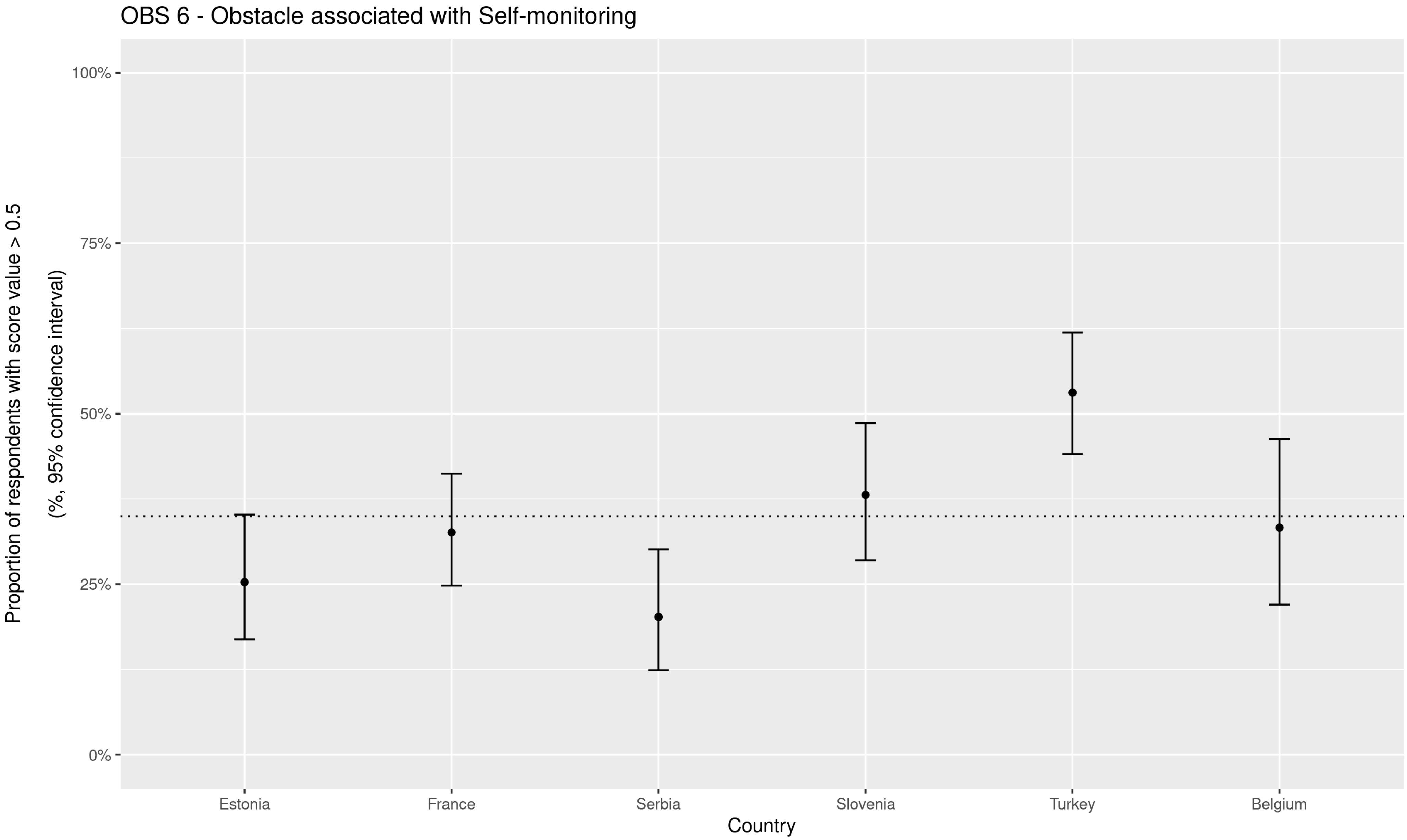
OBS 6 - Obstacle associated with Self-monitoring
Proportion of respondents with score value > 0.5
(%, 95% confidence interval)
100%
75%
50%
25%
0%
Estonia
France
Serbia
Slovenia
Turkey
Belgium
Country

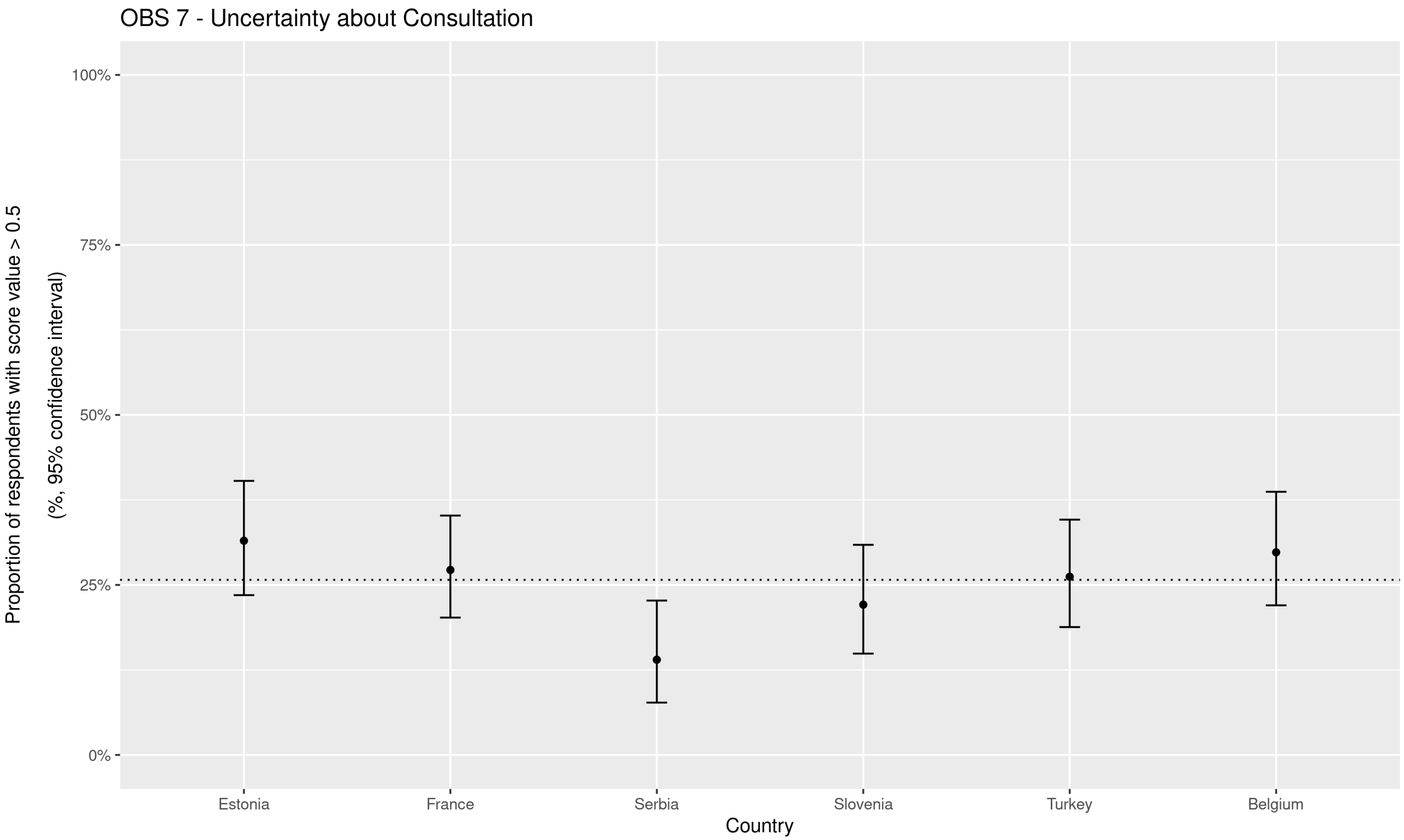
OBS 7 - Uncertainty about Consultation
Proportion of respondents with score value > 0.5
(%, 95% confidence interval)
100%
75%
50%
25%
0%
Estonia
France
Serbia
Slovenia
Turkey
Belgium
Country

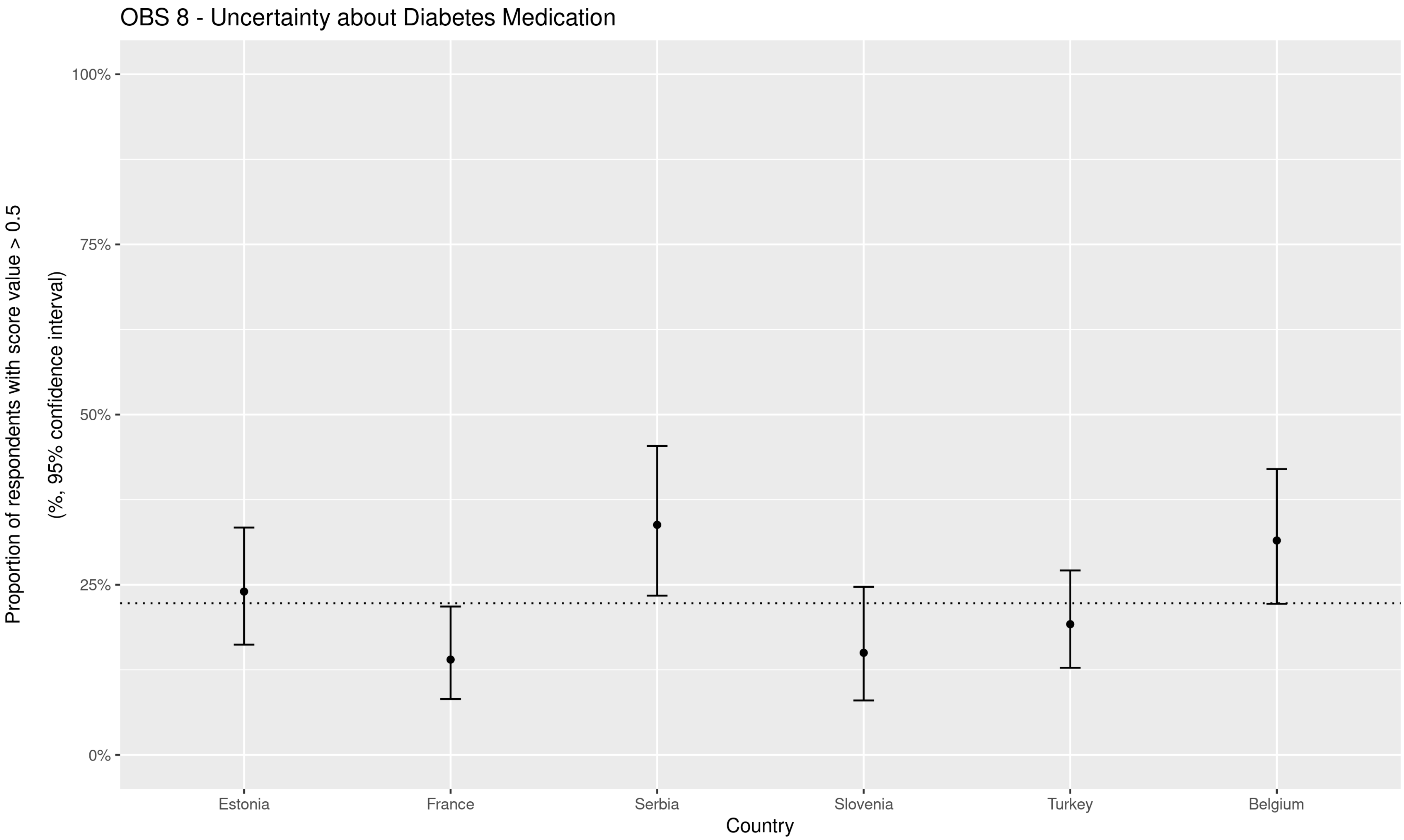

OBS 8 - Uncertainty about Diabetes Medication
Proportion of respondents with score value > 0.5
(%, 95% confidence interval)
100%
75%
50%
25%
0%
Estonia
France
Serbia
Slovenia
Turkey
Belgium
Country

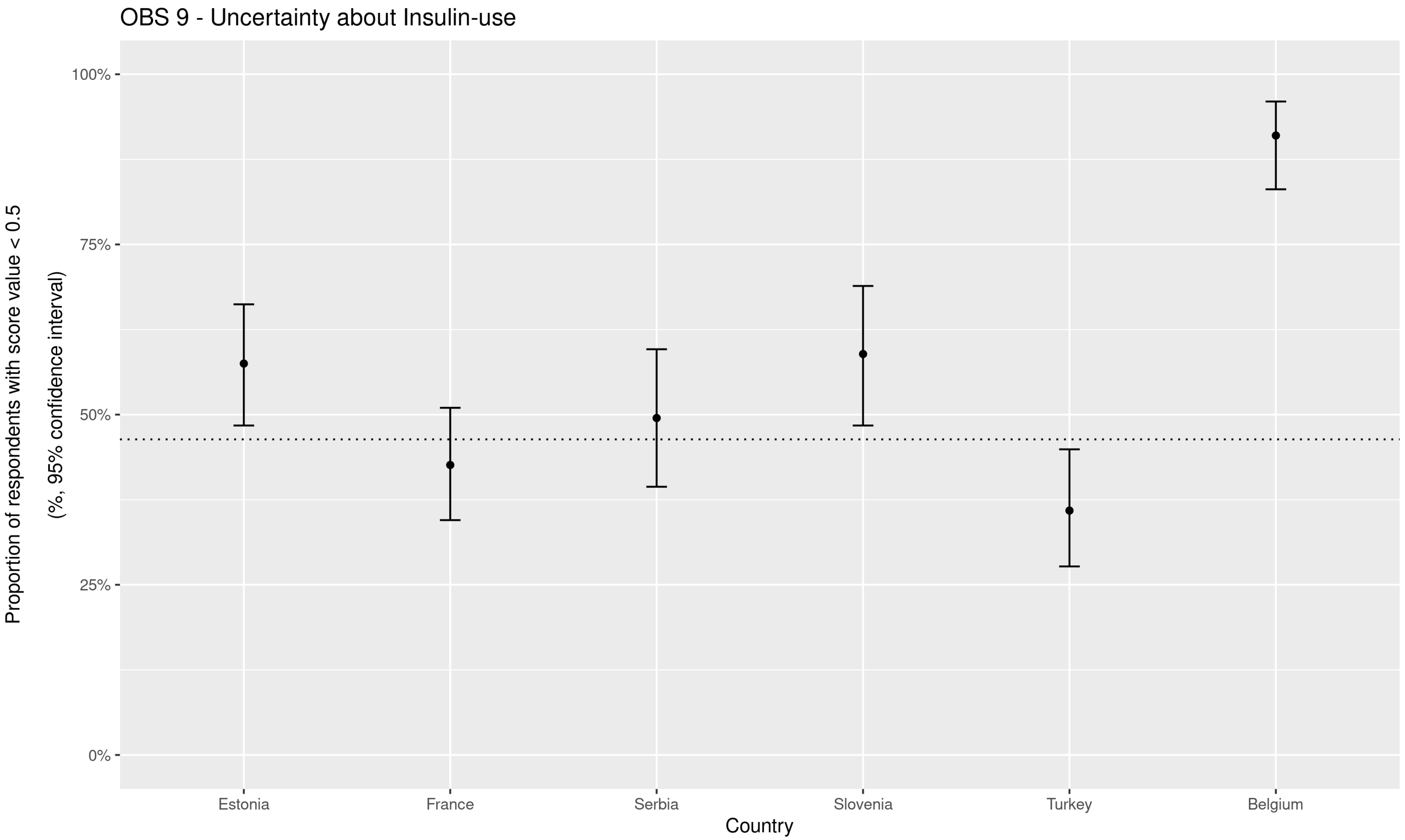

OBS 9 - Uncertainty about Insulin-use
Proportion of respondents with score value < 0.5
(%, 95% confidence interval)
100%
75%
50%
25%
0%
Estonia
France
Serbia
Slovenia
Turkey
Belgium
Country
